# Resting-State Functional Connectivity and Cognitive Impairment in Children with Perinatal Stroke

**DOI:** 10.1155/2016/2306406

**Published:** 2016-12-15

**Authors:** Nigul Ilves, Pilvi Ilves, Rael Laugesaar, Julius Juurmaa, Mairi Männamaa, Silva Lõo, Dagmar Loorits, Tiiu Tomberg, Anneli Kolk, Inga Talvik, Tiina Talvik

**Affiliations:** ^1^Department of Radiology, University of Tartu, Tartu, Estonia; ^2^Radiology Clinic of Tartu University Hospital, Tartu, Estonia; ^3^Department of Pediatrics, University of Tartu, Tartu, Estonia; ^4^Children's Clinic of Tartu University Hospital, Tartu, Estonia; ^5^Department of Development and Rehabilitation Centre of Children and Adolescents, Children's Clinic of Tartu University Hospital, Tartu, Estonia; ^6^Institute of Psychology, University of Tallinn, Tallinn, Estonia; ^7^Department of Pediatric Neurology, University of Helsinki and Helsinki University Hospital, Helsinki, Finland; ^8^Department of Neurology and Neurorehabilitation, Children's Clinic of Tartu University Hospital, Tartu, Estonia; ^9^Tallinn Children's Hospital, Tallinn, Estonia

## Abstract

Perinatal stroke is a leading cause of congenital hemiparesis and neurocognitive deficits in children. Dysfunctions in the large-scale resting-state functional networks may underlie cognitive and behavioral disability in these children. We studied resting-state functional connectivity in patients with perinatal stroke collected from the Estonian Pediatric Stroke Database. Neurodevelopment of children was assessed by the Pediatric Stroke Outcome Measurement and the Kaufman Assessment Battery. The study included 36 children (age range 7.6–17.9 years): 10 with periventricular venous infarction (PVI), 7 with arterial ischemic stroke (AIS), and 19 controls. There were no differences in severity of hemiparesis between the PVI and AIS groups. A significant increase in default mode network connectivity (FDR 0.1) and lower cognitive functions (*p* < 0.05) were found in children with AIS compared to the controls and the PVI group. The children with PVI had no significant differences in the resting-state networks compared to the controls and their cognitive functions were normal. Our findings demonstrate impairment in cognitive functions and neural network profile in hemiparetic children with AIS compared to children with PVI and controls. Changes in the resting-state networks found in children with AIS could possibly serve as the underlying derangements of cognitive brain functions in these children.

## 1. Introduction

Perinatal stroke leads to congenital hemiparesis [1–6]; however, these children may also have neurocognitive deficits, language impairment, behavioral disorders, and epilepsy [2–4, 7–14]. Previous outcome studies of perinatal stroke have been mainly focused on an isolated clinical function: either motor function [1, 2, 4, 7, 9, 15] or cognitive function [3, 5, 8, 13–16].

Perinatal ischemic stroke is a group of heterogeneous conditions in which there is a focal disruption of the cerebral blood flow secondary to arterial or venous thrombosis or embolization during the perinatal period [17]. Based on clinical-radiographical findings, there are two main subtypes of perinatal stroke: arterial ischemic stroke (AIS) and periventricular venous infarction (PVI) [3, 4, 9, 18]. The AIS involves arterial occlusion mostly in the middle cerebral artery (MCA) territory [3, 4, 9, 18], comprising large areas in the parietal, temporal, and frontal cortex and/or in the basal ganglia including the corticospinal tract [2]. The PVI, in contrast, comprises purely subcortical areas, predominantly the periventricular white matter, but also other parts of the descending corticospinal tract [3, 9]. In both types of vascular injury, damage of the corticospinal tract leads to hemiparesis [1, 2, 6].

Previous data about long-term cognitive development mostly apply to children with perinatal AIS [7–10, 13, 14, 16]; however, results vary to a great extent. Cognitive deficit in children with presumed PVI has received less attention and these children are usually investigated together with children with presumed AIS [4, 7, 14].

Task-based functional magnetic resonance imaging (fMRI) is used to investigate separate functions like motor [19] and language [20, 21] functions in stroke children. The fMRI has revealed the functional plasticity of the brain in the case of focal brain damage in perinatal stroke patients [19, 21]. However, task-based studies present several challenges for stroke patients, including motor task-related motion artifacts, inconsistent performance, mirror movements, or individual ability to perform the task altogether [22]. Furthermore, although structural damage from stroke is focal, remote dysfunction can occur in regions connected to the area of lesion [23].

Resting-state fMRI (rs-fMRI) is acquired in the absence of a task, which allows exploring the global functional organization of the brain and how it is altered in brain damage. It is now well established that many resting-state networks (RSN) are robust, that is, consistent across subjects, and involve the sensory (visual, auditory, and somatosensory) and motor regions of the brain, as well as a number of associative “control” networks (default, dorsal stream, frontoparietal, and ventral stream) [24, 25]. A framework based on connectivity and neural communication across the brain regions provides us with a view of the brain as organized in an ensemble of functional networks in adults [25, 26] and in children [27, 28]. However, there exist differences in functional connectivity in global RSN between infants and young children, on one hand, and adults, on the other, which are due to brain developmental progression in regional and network specialization [28, 29]. During childhood, changes occur in the hierarchical and regional organization of brain connectivity, and functional connections between distant regions become stronger with advancing maturation [30]. The process of network maturation appears to be parallel with the progress of behavioral maturation and sensorimotor development precedes the development of the systems underlying higher cognition [31]. Furthermore, certain networks, such as the default mode network (DMN), are only slightly functionally connected in childhood but increase in connection strength over time until they are fully developed by adulthood [29]. Thus, an early childhood stroke that affects immature connections might have a stronger impact on functional reorganization compared to a stroke that affects more mature networks [32].

The relationship between functional connectivity, sensory deficits, and structural abnormality remains poorly understood [33]. However, to our knowledge, rs-fMRI data for children with stroke are limited and refer only to the somatosensory system. Dinomais et al. [33] have investigated rs-fMRI in perinatal stroke patients with cortical and periventricular lesions and demonstrated a relationship between functional connectivity and somatosensory impairment. Children who had lesions in the MCA territory displayed significantly less functional connectivity in the somatosensory cortex than children with periventricular lesions [33]. Recently, a small group of children with spastic cerebral palsy [34] and another group of hemiplegic cerebral palsy after cortical and subcortical damage [35] were investigated with rs-fMRI without data about the vascular origin of the damage. Saunders [36] found differences in the motor network in patients with perinatal AIS and PVI compared to controls and suggested that in these children extensive plasticity in the brain occurs after experiencing a stroke, which consequently has an effect on the functional connections between the areas of the brain at rest. There were also significant differences in plasticity between the AIS and PVI groups, suggesting that the functional reorganization of the motor function is different in each of these groups [36].

The aim of the study was to identify differences in resting-state networks and cognitive development in children with perinatal AIS and PVI and to compare the obtained data with the corresponding data for healthy controls.

## 2. Methods

### 2.1. Patients

The Estonian Pediatric Stroke Database [5] contains data for 80 children with perinatal stroke, initially collected for an epidemiological study (1994–2003) and prospectively updated through 2015. All radiological images in Estonia are archived in a single all-Estonian Picture Archiving System.

Of the 80 children with perinatal stroke, those with neonatal sinovenous thrombosis (*n* = 4), neonatal hemorrhagic stroke (*n* = 7), or inadequate magnetic resonance imaging (*n* = 2) were excluded.

Ischemic stroke was classified as AIS or PVI using the criteria based on a previous study by Kirton and coworkers [4] and modified by Ilves and coworkers [3, 21]. Patients were only considered eligible for our preliminary study when (a) they had documented unilateral left-hemisphere AIS or PVI (for the sake of homogeneity in the study as perinatal stoke affects more often the left side according to previous studies [18]) but also in our database (68%); (b) they were aged 7–17; and (c) they were able to remain still for about 45 minutes without sedation and to follow instructions during the MRI investigation.

Of the 67 patients with perinatal ischemic stroke, 22 had unilateral left-side PVI and 24 had unilateral left-side AIS. The parents were contacted by phone and were asked to participate in the outcome study. Also, the parents were enquired about the child's ability to undergo the MRI investigation without sedation; otherwise only studies of cognitive and motor outcome were applied. Eleven children with PVI, 10 children with AIS, and 25 ages and sex matched healthy voluntary controls without contraindications for MRI agreed to participate in the rs-fMRI investigation. The control children were recruited from among children of the hospital staff members and their acquaintances and from among their classmates and friends attending regular school without learning difficulties. Written informed consent was obtained from the parents and from the children aged seven years or older for participation in the study in accordance with the Declaration of Helsinki. The study was approved by the Ethics Review Committee on Human Research, University of Tartu (Protocol no. 170/T-17 from 28.04.2008).

One child (1/11) with PVI, three with AIS (3/10), and six controls (6/25) were excluded from the analysis due to artifacts in the acquired MRI sequences or due to a shorter than planned investigation time.

The final rs-fMRI analysis included 36 children, among them 10 with PVI (age range 7.6–15.9 years, 3 boys), 7 with AIS (age range 10.4–17.4 years, 5 boys), and 19 age and sex matched controls (age range 8.1–17.9 years, 9 boys) without significant differences in age or sex between the groups. The individual demographic and neuroimaging data of the children with PVI and AIS are presented in Table 1.

### 2.2. Neurodevelopmental Assessments and Analysis

All children with PVI had been symptom-free after birth and had received the diagnosis of presumed perinatal stroke after 28 days of life. Four children with AIS were diagnosed after birth and three were diagnosed beyond the neonatal period (presumed AIS).

Clinical evaluation of stroke patients was made by pediatric neurologists (R L, S L) according to Pediatric Stroke Outcome Measurement (PSOM) [37]. The PSOM is a disease-specific outcome measure for children with stroke and comprises 115 test items. It yields a deficit severity score ranging from 0 to 2 (0: no deficit, 0.5: mild deficit, normal function, 1: moderate deficit, impaired function, and 2: severe deficit, missing function) for five subscales: right sensorimotor, left sensorimotor, language production, language comprehension, and cognitive/behavioral performance. Hemiparesis was diagnosed in children who had abnormal tone and reflexes associated with moderate or severe sensorimotor impairment (impaired or missing function), that is, hemiplegic cerebral palsy and congenital hemiparesis.

Cognitive performance was evaluated by a clinical psychologist (M. M.) who was blinded to the data for the stroke vascular subgroup or any rs-fMRI data, using the Kaufman Assessment Battery for Children, Second Edition (K-ABC II) [38]. The battery comprises (a) Fluid-Crystallized Index, a general measure of cognitive ability that includes acquired knowledge; (b) Mental-Processing Index, a measure of mental processing ability that excludes measures of acquired knowledge; and (c) Nonverbal Index, a general measure of nonverbal abilities. In addition, standard scores for five subscales (sequential and simultaneous processing, learning, planning, and knowledge) are provided. The range of possible scores is from 40 to 160 (mean 100, SD 15).

**Figure 1:**
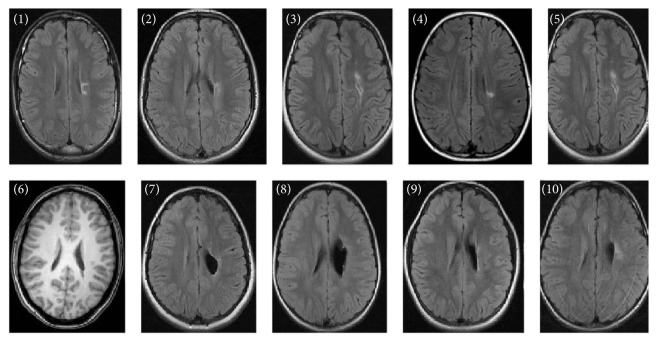
Anatomical fluid attenuated inversion recovery sequence images (patient (6) with T1 weighted image) for each of the patients with periventricular venous infarction according to patient number in Table 1. The single axial slices display maximum lesion volume. The individual injury patterns are detailed in Table 1.

**Figure 2:**
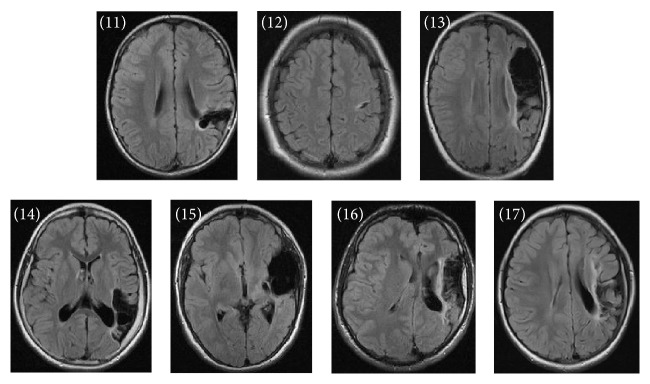
Anatomical fluid attenuated inversion recovery sequence images for each of the patients with arterial ischemic stroke according to patient number in Table 1. The single axial slices display maximum lesion volume. The individual injury patterns are detailed in Table 1.

Ischemic lesions were classified by the location and extent as described earlier [3, 22]. Among the children with PVI, five had small periventricular white matter damage in one lobe (patients (1) to (5)), one child had unilateral ventricular enlargement (patient (6)), and four had large left-side periventricular porencephalic damage involving the periventricular area in several lobes (patients (7) to (10)) (Figure 1). Among the children with AIS, there were two with a cortical stroke involving one lobe only (patients (11) and (12)) and five with a large cortical stroke involving several lobes and/or basal ganglia (patients (13) to (17)) (Figure 2). All arterial strokes were located in the MCA region: two in the proximal MCA territory, two in the distal MCA territory, and two in the posterior trunk of MCA and one in the anterior trunk of MCA (Table 1). There were no differences in the size of stroke (defined by involvement of one or several lobes) between the PVI and the AIS children (*p* = 0.33) (Table 1).

### 2.3. MRI Acquisition

The MRI data were acquired with the Philips 3-T Achieva MR scanner using the 8-channel SENSE head coil 3.0T/8ch (Philips Medical Systems, Best, The Netherlands). The scans were performed without sedation or medication; the participants were asked to stay awake and keep their eyes open.

The scans were acquired using a fixed imaging protocol after the acquisition of an anatomical scan. The T1 weighted slices of the whole head were obtained using a 3D fast field echo sequence (TR = 8.2 ms, TE = 3.8 ms), with a field of view of 256 × 256 mm and an isotropic voxel size of 1 mm. To describe resting-state activity in the brain, 120 volumes of 50 axial T2^*∗*^-weighted slices of the whole head were acquired using a fast field echo single shot EPI-BOLD sequence (TR = 3000 ms, TE = 35 ms), with a field of view of 230 × 230 mm and a voxel size of 3 mm isotropic.

The rs-fMRI data were visually inspected for motion and other imaging artifacts; entire scans were excluded from further analysis or, when possible, the scan was rescheduled. During motion correction, the maximum calculated absolute mean displacement was 0.78 mm and the maximum relative mean displacement was 0.29 mm [39].

### 2.4. Data Preprocessing

The analysis of rs-fMRI was made by a single investigator (N. I.) who was blinded to any clinical information or to the results of the cognitive tests, using the Multivariate Exploratory Linear Decomposition into Independent Components (MELODIC) tool, version 3.14 employing FSL from the FMRIB Software Library (https://www.fmrib.ox.ac.uk/fsl/) [40].

The following preprocessing workflow consisted of the following steps: (a) discarding of the first 2 volumes from each subject for signal stabilization; (b) motion correction using MCFLIRT [41]; (c) brain extraction of BOLD images; (d) spatial smoothing with FWHM 6 mm; (e) high-pass temporal filtering for 150 seconds.

Blood-oxygen level dependent (BOLD) volumes were registered to the T1 weighted structural volumes with 6 degrees of freedom using a boundary-based registration algorithm. Subsequently, the structural images were registered to the MNI-152 standard space with 12 degrees of freedom (T1 standard brain averaged over 152 subjects; Montreal Neurological Institute, Montreal, QC, Canada) [41, 42]. Normalized 4D datasets were subsequently resampled to 4 mm isotropic voxels.

### 2.5. Extracting Resting-State Networks

The data of the resting-state functions for both the study and control groups were temporarily concatenated and analyzed using probabilistic independent component analysis (PICA) [27]. The concatenated dataset was decomposed into 30 independent components. The components were visually evaluated and compared to previous literature data [24, 25, 27, 43–45] and 13 out of the 30 components were identified as anatomically and functionally relevant separate resting-state networks. The other 17 components reflected artifacts. The criteria for inclusion were signal within a low frequency range of 0.1–0.01 Hz [46, 47], location of connectivity patterns mainly in gray matter, and presence of coherent voxel clusters [48].

The subject-specific statistical maps of all RSN were created using a dual-regression tool from the FMRIB Software Library [24, 49] to test for differences in the identified components between the AIS, PVI, and control groups. Subsequently, groupwise comparison of RSN was carried out using a randomized, permutation-testing tool Version 2.9 from FSL. For each resting-state network, threshold-free cluster enhancement (TFCE) [50] was performed. The resulting statistical maps were thresholded at *p* ≤ 0.05 and at *p* ≤ 0.01 (TFCE corrected for familywise errors) for revealing group main effects. Inference was only carried out on the subject specific* z*-maps of 13 relevant RSNs. Between-group effects were thresholded controlling for local false discovery rate (FDR) [49] at *q* ≤ 0.1 to reduce susceptibility to type 1 errors when testing multiple resting-state networks.

Statistical evaluation was performed with the statistical package SAS Version 9.1 (SAS Institute INC, Cary, NC). Prior to further analysis, normality of the data was evaluated using the Kolmogorov-Smirnov criterion. To compare the proportions, the Chi-square test and Fisher's exact test (when the expected values were <5) were used. The nonparametric Mann-Whitney *U*-test was employed to compare the groups of AIS and PVI. Values are presented as means with the 95% confidence interval. The alpha level used to determine significance is *p* < 0.05. All *p* values are two-sided.

## 3. Results

### 3.1. Neurodevelopmental Outcome

The clinical findings and the data of cognitive functions for the children with PVI and AIS are presented in Tables 1 and 2 and the radiological findings are presented in Figures 1 and 2. Total PSOM score was abnormal for all stroke children. The children with AIS had significantly higher total PSOM scores compared to the children with PVI (*p* = 0.0486). All children had mild to severe sensorimotor deficit. However, 4/7 (57%) of the children with AIS and 8/10 (80%) of the children with PVI had moderate to severe hemiparesis; the difference between the PVI and AIS groups was not statistically significant (*p* = 0.59).

Most children with AIS (5/7, 71%) and only one child (1/10, 10%) with PVI had cognitive deficit according to PSOM (*p* = 0.035).

**Figure 3:**
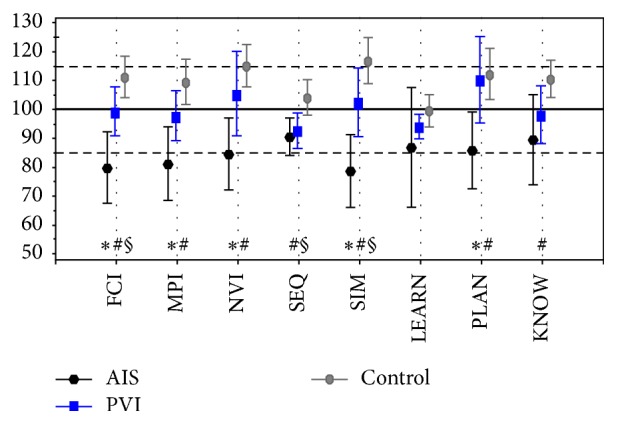
Mean with 95% CI Kaufman Assessment Battery for Children, Second Edition index with the subscale scores for children with periventricular venous infarction (PVI), arterial ischemic stroke (AIS), and controls. The FCI stands for Global Fluid-Crystallized Index (includes all subscales); MPI stands for Mental Processing Index (excludes acquired knowledge); NVI stands for Nonverbal Index; SEQ stands for Sequential Processing; SIM stands for simultaneous processing; LEARN stands for learning; PLAN stands for planning; KNOW stands for knowledge. Standard mean (SD) value for the battery is 100 (85–115). ^*∗*^ *p* < 0.05 AIS versus PVI. ^#^ *p* < 0.01 AIS versus controls. ^§^ *p* < 0.05 PVI versus controls.

According to the Kaufman Assessment Battery for Children, the children with AIS received significantly lower scores (Figure 3) in all three general ability indexes than the children of the PVI group: FCI (mean 79.7 versus 99.2, *p* = 0.013), MPI (mean 81.1 versus 97.7, *p* = 0.017), and NVI (mean 84.4 versus 105.3, *p* = 0.022). The PVI group outperformed the AIS group also in the subscale scores, while the results were significantly better for the children with PVI in simultaneous information processing (mean 102.3 versus 78.6; *p* = 0.015) and in planning ability (mean 110.2 versus 85.7; *p* = 0.017). The children with AIS performed significantly lower than the controls in all general ability and subscale indexes, except for learning. The overall cognitive development of the children with PVI in our study remained roughly within a normal range. However, children with PVI got lower results, compared to the control group in one general ability score (FCI), and in two subscales (simultaneous and sequential information processing) (Figure 3).

None of the 10 PVI children had epilepsy; however, 5/7 children in the AIS group had epilepsy and received antiepileptic medication (*p* = 0.0034).

### 3.2. Resting-State Functional Connectivity

**Figure 4:**
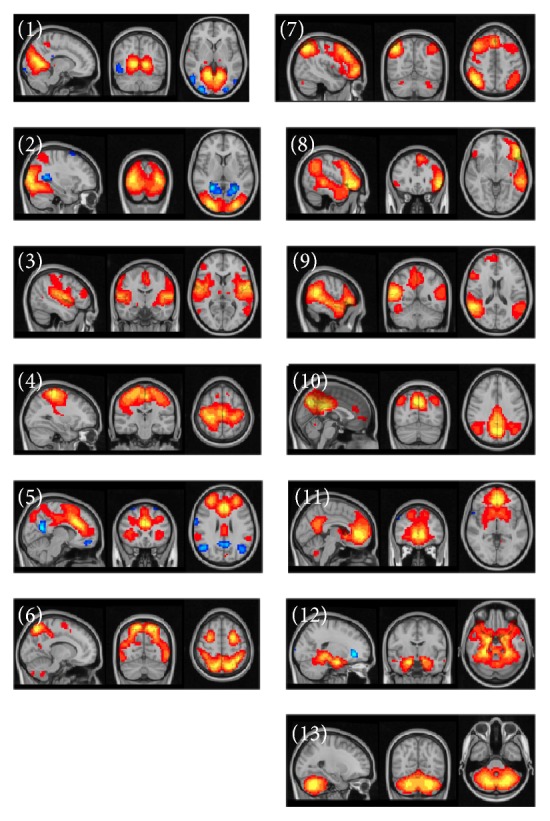
Resting-state networks estimated with ICA. The images are *z* statistics of the concatenated dataset for the controls and the stroke patients decomposed into the independent network components: primary visual (1), lateral visual cortex (2), auditory cortex (3), sensory-motor cortex (4) network associated with salience processing (5), task positive network involved in higher-order cognition and attention (6), networks implicated in working memory and cognitive attentional processes as right lateral network (7), and left lateral frontoparietal network (8), ventral stream ventral attention system (9), posterior component of the default mode network in the precuneus and parietal regions (10), anterior component of the default mode network in the frontal pole and precuneus (11), medial temporal/the hippocampus amygdala complex (12), and cerebellar network (13).

Thirteen functionally relevant RSN were found using group PICA (Figure 4). Such networks have been described in previous studies using a similar methodology for adults [24–26, 51] and for children [27, 28, 43]. According to the present study, the networks were stable across the participants of the AIS, PVI, and control groups. All of these 13 networks were found with independent PICA analysis for each of the PVI, AIS, and control groups.

### 3.3. Differences in Functional Connectivity between the Stroke and Control Groups

**Figure 5:**
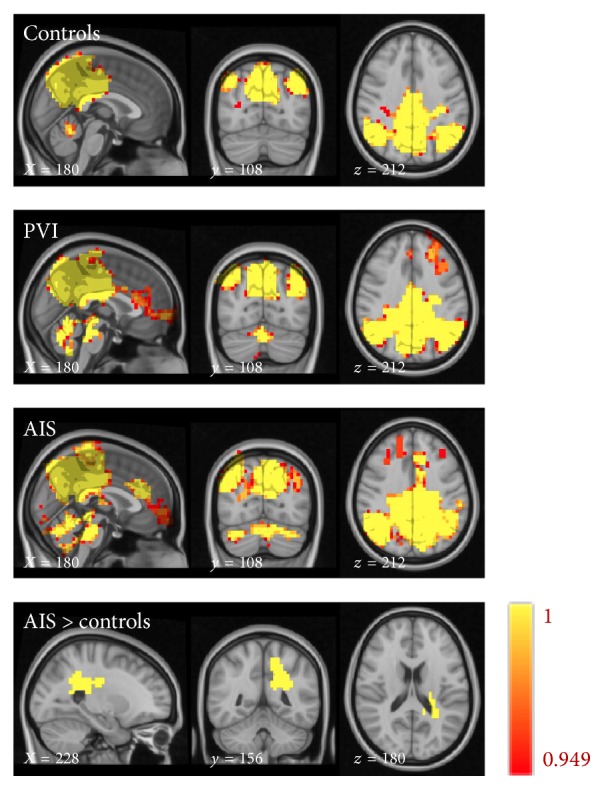
Connectivity maps of the posterior precuneus part of the default mode network for the controls, periventricular venous infarction (PVI), arterial ischemic stroke (AIS), and a map of the areas with increased connectivity in AIS versus control (FDR 0.1). Color map from 0.949 (red) to 1 (yellow).

All 13 RSN networks were included in group-level analyses. Testing of the main effects of the group on the subject specific* z*-maps of these networks (all *p* ≤ 0.05 and also *p* ≤ 0.01 TFCE corrected for familywise errors) showed significantly increased functional connectivity in the posterior and anterior components of DMN and in the task positive and medial temporal networks in the patients with AIS (*p* < 0.01), compared to the controls. These networks, except for the anterior component of DMN (*p* < 0.05), were even increased in AIS compared to PVI (*p* < 0.01), corrected for familywise errors. However, after FDR correction (local FDR-corrected at *q* ≤ 0.1), significantly increased functional connectivity was only found in the DMN posterior component in the left periventricular area of the AIS patients versus the controls (Figure 5).

The control group showed increased functional connectivity in the primary visual, salience, task positive, cerebellum, and sensorimotor networks compared to the AIS group (*p* < 0.01); however, after FDR corrected analysis at *q* ≤ 0.1, the difference was not significant.

The PVI group showed increased functional connectivity in the medial visual, auditory, salience, ventral stream, and cerebellum networks compared to the patients with AIS (*p* < 0.01); however, after FDR corrected analysis at *q* ≤ 0.1 level, the difference was not significant.

There were no statistical differences between the children of the PVI and control groups in RSN.

## 4. Discussion

We report differences in the rs-fMRI networks and cognitive functions in children with left-hemisphere perinatal stroke of different vascular origin. More severe dysfunctions in the rs-fMRI RSN networks and cognitive functions occurred in the AIS compared to the PVI children. These results provide a preliminary insight into large-scale brain network dysfunction that may be the underlying cause of the various motor, cognitive, and behavioral problems in patients with perinatal stroke.

### 4.1. Neurodevelopmental Outcome

The children with AIS had significantly higher PSOM scores compared to the PVI children. Depending on vascular origin, different pathogenetic mechanisms behind AIS and PVI are responsible for the location of brain damage and outcome [3, 4]. In AIS, cortical-subcortical involvement is the most prominent location of damage; in PVI, the main location of damage is the periventricular white matter while the cortical areas are spared [4]. However, the corticospinal tract is often damaged, although in different locations, in the case of both types: 24–60% of cases in AIS [1, 2] and up to 80% of cases in PVI [9].

We found that although motor outcome was similar in children from both the AIS and PVI groups, the measures of cognitive ability based on K-ABC-II scores were lower in AIS compared to PVI. An earlier study by Westmacott and coworkers [14] also suggested that motor function was not linearly related to cognitive outcome and PSOM motor scores were not correlated with IQ measures. Some studies suggest, however, that overall cognitive development in perinatal stroke falls roughly within a normal range [10, 13–15], although children with PVI and AIS are often studied in one group.

Ricci and coworkers [13] have found that only one-third of children show cognitive deficit after perinatal stroke. In some other studies, children with neonatal AIS have significantly lower scores for working memory and processing speed compared to the normative population [1]. This is also the case with the measures of general cognitive ability, verbal functioning, inhibitory control and working memory [8], or the measures affecting complex cognitive skills as abstract reasoning [14]. Our data confirmed that, according to K-ABC-II, the children of the AIS group had significantly lower scores for general ability than the children of the PVI group. In addition, the former were less successful in the tasks that required higher cognitive abilities such as planning or simultaneous visual processing.

Cognitive problems in the children with PVI, all with presumed stroke, were less pronounced and only 1 of the 10 children in the study group had some mild cognitive problems. In an earlier study investigating both presumed stroke with AIS and PVI, the proportion of children with presumed perinatal stroke who had cognitive or behavioral deficits was 29% [4]. As in our study, the children with PVI had less behavioral and visual deficits than the children with a large proximal MCA stroke but they had more spasticity than the children with stroke in the anterior trunk of MCA [4]. However, adverse cognitive and behavioral outcomes were more strongly correlated with cortical involvement compared to periventricular involvement in presumed perinatal stroke [4].

### 4.2. Rs-fMRI Investigations

We found significant global derangements in the cognitive networks at rs-fMRI and in the cognitive function tests in the children with AIS compared to the PVI and control groups. Global neural network dysfunction can serve as a possible basis for derangements of complex cognitive functions and behavior as has also been reported in earlier stroke outcome studies [4, 10, 14]. The RSN of the children with PVI was not different from the corresponding measures for the control group.

Large-scale networks were identified in which the patients with AIS showed significantly increased functional connectivity, compared to the controls and the children with PVI, in the posterior precuneus part and the anterior frontal part of DMN, in the medial temporal component of DMN, and in the task positive network (*p* < 0.01). These networks were even increased in AIS compared to PVI (*p* < 0.05). However, after FDR correction, significantly increased functional connectivity was only found in posterior precuneus part of DMN in the AIS children compared to the controls. The DMN is deactivated during demanding cognitive tasks and is involved in episodic memory processes and self-referential mental representations [45]. The task positive network is involved in higher-order cognition and attention [45].

Earlier studies have found that most networks in children, in particular those supporting the basic motor function and sensory related processing, had a robust functional organization similar to mature adult patterns [27, 28, 43]. In contrast, DMN and the other RSN involved in the higher-order cognitive functions had immature characteristics, revealing incomplete and fragmented patterns, which indicates less developed functional connectivity, in infants [28] aged 5 to 8 years [27] and even at the age of 10–13 years [43]. A major difference between adults and children is the decomposition of DMN into several independent subsystems in children, composed of the bilateral posterior cingulate, the precuneus, the inferior parietal cortex, and the ventromedial prefrontal cortex, which are associated with the medial temporal regions [27, 29]. At the same time, the posterior precuneus region of the DMN network serves as the main hub within DMN [27]. The DMN has been found to be only weakly functionally connected in childhood but increases in connection strength over time. Therefore, an early childhood stroke that affects immature connections might have a stronger impact on functional reorganization than a stroke that affects more mature networks [29]. However, there exists also the hypothesis that damaged areas continue to support the performance of tasks involving them and the typical functional connections of these regions play an important role in preservation of normalcy after early perinatal stroke [52]. In our study significant changes in DMN and especially in the posterior precuneus part of DMN, which is the main hub within DMN, were found in the children with AIS who had also serious cognitive problems. Also, were found in the AIS and control children some derangements of the task positive network, which is part of the attention control networks. The attention control networks have generally immature characteristics in young age groups [27].

### 4.3. Location of Stroke and rs-fMRI Networks

Cortical involvement of damage in AIS compared to PVI with mainly periventricular damage can explain differences in network dysfunction. Increased connectivity of the networks outside the region of primary damage (DMN) can be a compensatory effect occurring after cortical brain damage in children with AIS due to brain's plasticity; in this case areas outside the lost tissue take over its functions. A similar finding was obtained by a task-based fMRI [20, 21]. The prefrontal regions, both ipsi- and contralateral to the lesion, were activated in patients but not in controls after language and visual search tasks, which may compensate for lost functions according to task-based fMRI [20]. Such contralateral activation of the language function is seen more often in younger stroke patients before the age of 2 years [21]. It is unknown whether the frontal task-based networks are activated due to alternative or compensational strategies [20].

## 5. Limitations of the Study

The small number of rs-fMRI examinations of perinatal stroke could limit the statistical power and generalizability of the study results. Still, our preliminary data were available for 10 children with PVI, which is close to the 12 cases of presumed PVI reported in a previous clinical-radiological study by Kirton and coworkers [4]. Although significant differences both in cognitive function and in the global networks were found in the children with AIS versus PVI, more clinical and rs-fMRI data, especially for right hemisphere strokes, would be needed to evaluate the predictive value of rs-fMRI results for establishment of cognitive deficit in these children.

On our stroke database and in the cohort of this study, the children with AIS had significantly more often epilepsy compared to the children with PVI. Epilepsy per se and/or antiepileptic treatment may have had an impact on the cognitive ability of the former. Earlier studies have also found that seizures are more frequent in children with cortical damage compared to periventricular damage in presumed perinatal stroke [4]. Cognitive impairments with lower performance in the intellectual and language measures have been found in children with perinatal stroke associated with seizures [4, 10, 11, 13, 15].

Although there were no gender differences in our stroke group compared to the controls, there were more boys in the AIS group, which could also influence the cognitive outcome. Males with perinatal stroke performed tests significantly more poorly than a matched group of females in overall intellectual ability and in reading and processing speed [7, 14]. It should be noted that as about 60% of the children with PVI in the Estonian Perinatal Stroke Database are female [3], it was difficult to recruit male subjects with PVI to the study.

Also, we had to exclude from the study the most severe cases when the mother thought that the child would not be able to follow instructions and stay still during MRI without sedation. This could have diminished the statistical power of the rs-fMRI data for the AIS children. However, most of such cases were encountered in the AIS group with bilateral asymmetrical involvement.

## 6. Conclusions

Our findings demonstrate differences in the cognitive function and in the neural network profile of children with hemiparesis with left-side AIS compared to children with left-side PVI and controls matched for age and sex. The study shows that as the location of damage is different in children with AIS and PVI, also the resting-state networks and cognitive outcome are different in these groups and children with AIS and PVI should not be analyzed together in outcome studies.

Changes in the resting-state networks found in children with AIS could possibly serve as an underlying dysfunction of cognitive brain functions in perinatal stroke patients. In order to better understand the value of derangements in the brain networks in perinatal stroke patients with cognitive and behavioral problems, further studies are needed. It is important to promote early diagnosis, treatment, and rehabilitation of these children, to improve their quality of life, as well as the quality of life of their families.

## Figures and Tables

**Table 1:** Demographic and neuroimaging data for the children with periventricular venous infarction and arterial ischemic stroke.

Patients number	Sex	Gestational age at birth	Presumed or neonatal stroke	Type of stoke	Age at the time of resting-state functional MRI	Lesion location (left)	Lesion size 1–5
(1)	M	40	Presumed	PVI	15.9 years	F	2
(2)	F	40	Presumed	PVI	7.6 years	F	2
(3)	F	42	Presumed	PVI	10.6 years	F	2
(4)	M	40	Presumed	PVI	13.4 years	F	2
(5)	F	38	Presumed	PVI	14.6 years	Th-F	2
(6)	F	36	Presumed	PVI	14.6 years	P	1
(7)	F	38	Presumed	PVI	9.7 years	BG-Th-F-P	4
(8)	M	37	Presumed	PVI	10.8 years	BG-Th-F-P	4
(9)	F	34	Presumed	PVI	12.7 years	BG-Th-F-P	4
(10)	F	40	Presumed	PVI	8.6 years	F-P	4
(11)	M	42	Neonatal	AIS/PT	10.5 years	Th-P	3
(12)	F	38	Presumed	AIS/AT	15.3 years	F	3
(13)	M	40	Neonatal	AIS/DMI	10.7 years	Th-F-P	5
(14)	M	41	Neonatal	AIS/PT	10.5 years	F-T	5
(15)	M	39	Presumed	AIS/PMI	14.1 years	BG-Th-F-P	5
(16)	M	40	Presumed	AIS/PMI	17.4 years	BG-Th-F-P-T	5
(17)	F	39	Presumed	AIS/DMI	16.3 years	Th-F-P-T	5

Type of stroke: PVI: periventricular venous infarction; AIS: arterial ischemic stroke; PT: posterior trunk of the medial cerebral artery (MCA); AT: anterior trunk of the MCA; PMI: proximal MCA; DMI: distal MCA.

Lesion location: BG: basal ganglion; Th: thalamus; F: frontal cortex; P: parietal cortex; T: temporal cortex; O: occipital cortex.

Lesion size grading system:

(1) ventricular dilatation or atrophy; (2) focal periventricular damage involving one lobe only; (3) focal cortical damage involving one lobe only; (4) focal periventricular damage involving multiple lobes; (5) focal cortical damage involving multiple lobes.

**Table 2:** Clinical data and data of cognitive function for the children with periventricular venous infarction and arterial ischemic stroke.

Patients number	Type of stroke	Severity of the right hemiparesis mild/moderate/severe	PSOM	Cognitive dysfunction no/mild/moderate/severe	Seizures Yes/no	FCI score	MPI score	NVI score
(1)	PVI	Mild	0.5	No	No	111	108	127
(2)	PVI	Severe	2.5	No	No	103	97	98
(3)	PVI	Moderate	1.5	No	No	88	86	88
(4)	PVI	Severe	5	Mild	No	73	75	69
(5)	PVI	Moderate	1.5	No	No	104	104	108
(6)	PVI	Moderate	1.5	No	No	109	119	144
(7)	PVI	Moderate	1	No	No	111	97	105
(8)	PVI	Moderate	2	No	No	95	97	100
(9)	PVI	Mild	1	No	No	99	101	113
(10)	PVI	Moderate	1	No	No	99	93	101
(11)	AIS/PT	Mild	2	No	Yes	96	95	102
(12)	AIS/AT	Moderate	1	No	No	89	89	94
(13)	AIS/DMI	Moderate	3.5	Mild	No	79	78	80
(14)	AIS/PT	Mild	3	Mild	Yes	84	80	82
(15)	AIS/PMI	Severe	8	Severe	Yes	53	54	59
(16)	AIS/PMI	Severe	2.5	Mild	Yes	79	92	87
(17)	AIS/DMI	Severe	3	Mild	Yes	78	80	87

Type of stroke: PVI: periventricular venous infarction; AIS: arterial ischemic stroke; PT: posterior trunk of the medial cerebral artery (MCA); AT: anterior trunk of MCA; PMI: proximal MCA; DMI: distal MCA.
